# Pathway Implications of Aberrant Global Methylation in Adrenocortical Cancer

**DOI:** 10.1371/journal.pone.0150629

**Published:** 2016-03-10

**Authors:** Christophe R. Legendre, Michael J. Demeure, Timothy G. Whitsett, Gerald C. Gooden, Kimberly J. Bussey, Sungwon Jung, Tembe Waibhav, Seungchan Kim, Bodour Salhia

**Affiliations:** 1 Translational Genomics Research Institute, Phoenix, AZ, United States of America; 2 NantOmics, LLC, Phoenix, Arizona, United States of America; 3 Department of Genome Medicine and Science, Gachon University School of Medicine, Incheon, 21565, Republic of Korea; 4 Gachon Institute of Genome Medicine and Science, Gachon University Gil Medical Center, Incheon, 21565, Republic of Korea; CEA - Institut de Genomique, FRANCE

## Abstract

**Context:**

Adrenocortical carcinomas (ACC) are a rare tumor type with a poor five-year survival rate and limited treatment options.

**Objective:**

Understanding of the molecular pathogenesis of this disease has been aided by genomic analyses highlighting alterations in TP53, WNT, and IGF signaling pathways. Further elucidation is needed to reveal therapeutically actionable targets in ACC.

**Design:**

In this study, global DNA methylation levels were assessed by the Infinium HumanMethylation450 BeadChip Array on 18 ACC tumors and 6 normal adrenal tissues. A new, non-linear correlation approach, *the discretization method*, assessed the relationship between DNA methylation/gene expression across ACC tumors.

**Results:**

This correlation analysis revealed epigenetic regulation of genes known to modulate TP53, WNT, and IGF signaling, as well as silencing of the tumor suppressor MARCKS, previously unreported in ACC.

**Conclusions:**

DNA methylation may regulate genes known to play a role in ACC pathogenesis as well as known tumor suppressors.

## Introduction

Adrenocortical carcinomas (ACC) are rare neoplasms that account for up to 0.2% of cancer deaths. The estimated incidence of the disease is 0.7 to 2.0 cases per million persons per year [1, 2]. This disease usually strikes adults in their 40-50s, but may also be seen in children, typically with a Tumor Protein p53 (*TP53*) germline mutation [3]. The only curative treatment is surgical removal of a localized tumor; many patients have metastatic disease at the time of diagnosis, which further limits their therapeutic options [4, 5]. In a study looking at almost 4000 cases of ACC between 1985 and 2005, Bilimoria *et al*. reported 26.5% presented with nodal metastasis and 11.3% presented with distant metastasis, leading to a five-year survival rate of 11.5% compared to 55.1% without distant metastasis [4]. The response rate to the generally accepted first-line chemotherapy regimen consisting of etoposide, doxibucin, cisplatin and mitotane is only 23% [6], making it clear that there is an urgent need for new therapies.

Genomic analyses are elucidating the molecular pathogenesis of ACC and exposing potential therapeutic targets. The most well studied and consistently observed genomic aberrations involve the *TP53* tumor suppressor gene, insulin-like growth factor type 2 (*IGF2*) signaling, and the Wingless-Type MMTV Integration Site Family (*WNT*) pathway. Several studies have clearly established a role for aberrant TP53 function, even though the reported mutation rate for the *TP53* in sporadic adult ACC is only approximately 16–27% [7–9]. Overexpression of *IGF2* is seen in at least 95% of ACC tumor samples studied [10], prompting clinical trials employing Insulin-Like Growth Factor 1 Receptor (IGF1R) inhibitors [11]. WNT pathway signaling is also aberrant, with activating somatic mutations of the β-catenin gene seen with a similar frequency of approximately 30% in both benign and malignant adrenal cortex tumors [12]. While genomic analyses have uncovered pathway perturbations in ACC, clinically actionable targets have remained elusive, leading one to conclude that there is a need for a deeper understanding of ACC tumorigenesis.

Investigation of epigenetic alterations in ACC is garnering interest. Global methylation analyses have demonstrated that distinct methylation patterns exist to distinguish ACC from benign tumors and/or normal adrenal tissue [13, 14]. Rechache *et al*. have demonstrated that metastatic ACC tumors showed a distinct methylation pattern compared to normal adrenal and primary ACC tumors, and malignant samples demonstrated global hypomethylation [14]. Barreau *et al*. identified a CpG island methylator phenotype (CIMP) in ACC associated with poor patient survival [15]. These global methylation analyses suggested hypermethylation of tumor suppressors such as cyclin-dependent kinase inhibitor 2A (*CDKN2A*), Deleted in Lung and Esophageal Cancer 1 (*DLEC1*), or *N-Myc* Downstream-Regulated Gene family member 2 (*NDRG2*) as a mechanism for reduced mRNA expression observed in ACC tumors compared to adenomas or normal adrenal tissue. More recently, an integrated genomic characterization identified two distinct molecular subgroups in ACC: C1A and C1B [7]. The C1A subgroup correlated with a poor patient prognosis, increased frequency of driver genetic mutations, distinct mRNA and miRNA clusters, and had a clear association with CIMP. Thus, methylation analyses have identified subgroups of ACC tumors with differential prognosis and suggested molecular alterations that might contribute to ACC pathogenesis; although, the full extent of the genes and pathways modulated by methylation in ACC remains unknown.

In this work, we sought to correlate DNA methylation changes with expression profiles in a set of ACC (n = 18 [17 tumors, 1 liver metastasis ACC_150]) and normal adrenal (n = 6) samples in order to better understand how methylation may result in the aberrant gene and pathway expression observed in ACC. Our study uses a non-linear correlation approach referred to as *the discretization method* [16] to assess DNA methylation/gene expression relationships within pathways, and reveals potential epigenetic regulation of genes involved in TP53, WNT, IGF2, and tumor suppressor gene signaling and/or stability. While other studies have reported DNA methylation changes in ACC in the past, our study is distinct in the manner in which we describe DNA methylation/gene expression associations to identify epigenetically regulated pathways of known importance to ACC.

## Materials and Methods

### Clinical Samples

The clinical samples used in this analysis represent a subset of samples previously described [8]. Briefly, a set of ACC flash frozen tumors and normal adrenal glands were collected at the Mayo Clinic (Rochester, Minnesota), the University Hospital Essen (Essen, Germany), the University of Calgary (Alberta, Canada), and Scottsdale Healthcare (Scottsdale, Arizona), as well as donated directly by patients through their community care settings; all samples were obtained under appropriate ethical procedures and written informed patient consent at the respective institutions. Normal adrenal glands were collected at the time of surgery for another indication, typically resection of a tumor of the kidney. The adrenal was taken en bloc, and the cortex was macrodissected from the medulla as best possible. Research materials for this study were obtained under protocols approved by the Western Institutional Review Board (WIRB # 20051769). The diagnosis of ACC was confirmed by review of the pathology report, and in most cases, by reexamination of the histopathology slides by an experienced endocrine pathologist.

### Gene Expression Profiling

The mRNA expression and statistical analysis of ACC and normal tissues was previously described [8]. Briefly, RNA was extracted from 100 mg samples of ACC tumors and normal adrenal tissue, amplified and reverse transcribed utilizing the MessageAmp II Biotin Enhanced Kit (Ambion Life Technologies Corp, Carlsbad, CA). Biotin-labeled cRNA was synthesized according to their standard protocol, followed by purification through provided cRNA Filter Cartridges. Labeled cRNA was fragmented and hybridized to Affymetrix U133 Plus 2 human genome arrays following the standard Affymetrix protocol (Affymetrix Inc., Santa Clara, CA). Scanning and washing was completed on the Fluidic Stations FS450 and the GeneChip^®^ Scanner 3000 with Workstation.

Array quality for ACC and the normal samples was assessed using the Affy QCReport package in Bioconductor and the R statistical language; all arrays passed the quality control metrics. All subsequent data normalization and statistical analysis was done using GenePattern (Broad Institute, www.broadinstitute.org) [17]. Expression array data was normalized by gcRMA with quantile normalization, and background subtraction after using the Expression File Creator [18]. Data were then floored at 5.5 using Preprocess Dataset, and filtered to remove 1) probes with more than 35 floored values and/or 2) probes where all values from one batch were floored while values from the other batch were not. Further batch effects were minimized using ComBat with the parametric option [19]. The expression data discussed in this publication have been deposited in NCBI's Gene Expression Omnibus and are accessible through GEO Series accession number GSE19776 (http://www.ncbi.nlm.nih.gov/geo/query/acc.cgi?acc=GSE19776).

### DNA Methylation Analysis

Global DNA methylation was evaluated using the Infinium^®^ HumanMethylation450^®^ BeadChip Array. (Illumina, San Diego, CA). Briefly, 1 μg of each DNA sample underwent bisulfite conversion using the EZ DNA Methylation Kit (Zymo Research, Irvine, CA) according to the manufacturer’s recommendation for the Illumina Infinium Assay. Bisulfite-treated DNA was then hybridized to arrays according to the manufacturer’s protocol. We used GenomeStudio V2011.1 (Illumina) for methylation data assembly and acquisition. Methylation levels for each CpG residue are presented as ß values, estimating the ratio of the methylated signal intensity over the sum of the methylated and unmethylated intensities at each locus. The average ß value reports a methylation signal ranging from 0 to 1, representing completely unmethylated to completely methylated values, respectively. Methylation data was preprocessed in R using the Illumina Methylation Analyzer (IMA) package [20]. Data preprocessing included background correction, probe scaling to balance Infinium I and II probes, quantile normalization, and logit transformation. A logit transformation converts otherwise heteroscedastic beta values (bounded by 0 and 1) to M values following a Gaussian distribution. Additionally, detection p-values >0.05 in 25% of samples, probes on X and Y chromosomes, and probes situated within 10 bp of putative SNPs were removed. Differential methylation analysis on logit-transformed values was performed to compare 18 ACC tumors to 6 normal adrenal samples in IMA. Wilcox rank test was conducted between ACC and normal samples and p-values were corrected by calculating the false discovery rate by the Benjamini-Hochberg method. Probes with adjusted *p-values* <0.05, and delta β values ≥0.2 or ≤-0.2 were considered statistically significant and differentially methylated. The methylation data discussed in this publication have been deposited in NCBI's Gene Expression Omnibus and are accessible through GEO Series accession number GSE77871 (http://www.ncbi.nlm.nih.gov/geo/query/acc.cgi?acc=GSE77871).

### Correlating DNA Methylation to Gene Expression by the Discretization Method

We used our newly reported non-linear *discretization method* by categorizing samples into different groups based on probe methylation levels to identify DNA methylation/expression correlations [16]. Gene expression data was available for 14 of the 18 samples analyzed for methylation changes. For each methylation probe, the 14 ACC samples were separated into *hypermethylated* (M) or *hypomethylated* (U) groups based on the degree of methylation differing from the average methylation levels of 6 normal adrenal samples. Samples with methylation levels > *μ* (mean methylation level of normal samples) were classified into the *M* group and samples with methylation levels < *μ* were classified as U for the given CpG locus interrogated by the probe. Every probe in the methylation array with a gene expression probe on the Affymetrix U133 Plus 2 human genome array was analyzed. Differential gene expression analysis, comparing samples that were categorized as M and U, was conducted with a *t*-test without assuming equal variance (Matlab software, www.mathworks.com). If a gene was differentially expressed (FDR-corrected *p-value* < 0.05) between samples in the M and U groups, CpG methylation was considered correlated to expression of that gene. A negative correlation was defined as the directionality of change for expression and methylation in opposite directions (e.g. hypermethylation and loss of expression, or vice versa). A positive correlation occurred when the directionality of change was the same between methylation and expression (e.g. hypermethylation and positive expression, or hypomethylation and reduced expression).

To validate our findings reported in S2 Table we used RNAseq and methylation data from 78 ACC samples generated by TCGA. Level 3 RNAseq data (TPM values) and methylation beta values were downloaded from TCGA Data Portal (https://tcga-data.nci.nih.gov). RNAseq data were log2 transformed: log2(TPM + 1), before further analysis. In level 3 TCGA methylation data, some values were measured as “NA” and those values were removed during differential analysis. We also did not include those methylation probes with < 2 samples in either hypo- or hyper-group in the analysis. The validation analysis was conducted by testing if the same methylation probes reported in S2 Table also had correlation with gene expression in the TCGA ACC dataset, when using the discretization method. In the current manuscript, we modified the discretization method first described in Jung *et al*. [16] to reflect the small number of samples. In this modified method, we discretized samples based on whether a sample was hypo or hypermethylated when compared to non-neoplastic adrenal tissue. For this, a delta beta value for each probe was calculated for each tumor’s methylation data when compared against the mean of the methylation data from the non-neoplastic adrenal tissue. In this binary discretization, a tumor sample was deemed hypomethylated or hypermethylated when its delta-beta value was negative or positive, respectively, compared to normal tissue. Since there were sufficient samples in the TCGA dataset, we also performed discretization according to the original method described (referred to here as ternary discretization) by Jung *et al*. [16]. Once the samples were grouped into U and M groups by either binary or ternary discretization, the expression values of genes with corresponding methylation probes were tested for differential expression using Welch’s t-test.

### Pathway Analysis

The gene list of interest was uploaded into IPA (Ingenuity^®^ Systems, Redwood City, CA) and the Core Analysis workflow was run with default parameters. The Core Analysis provides an assessment of significantly altered pathways, molecular networks, and biological processes represented in the samples' gene list.

## Results

### Global Methylation Patterns in ACC versus Normal Adrenal

**Fig 1:**
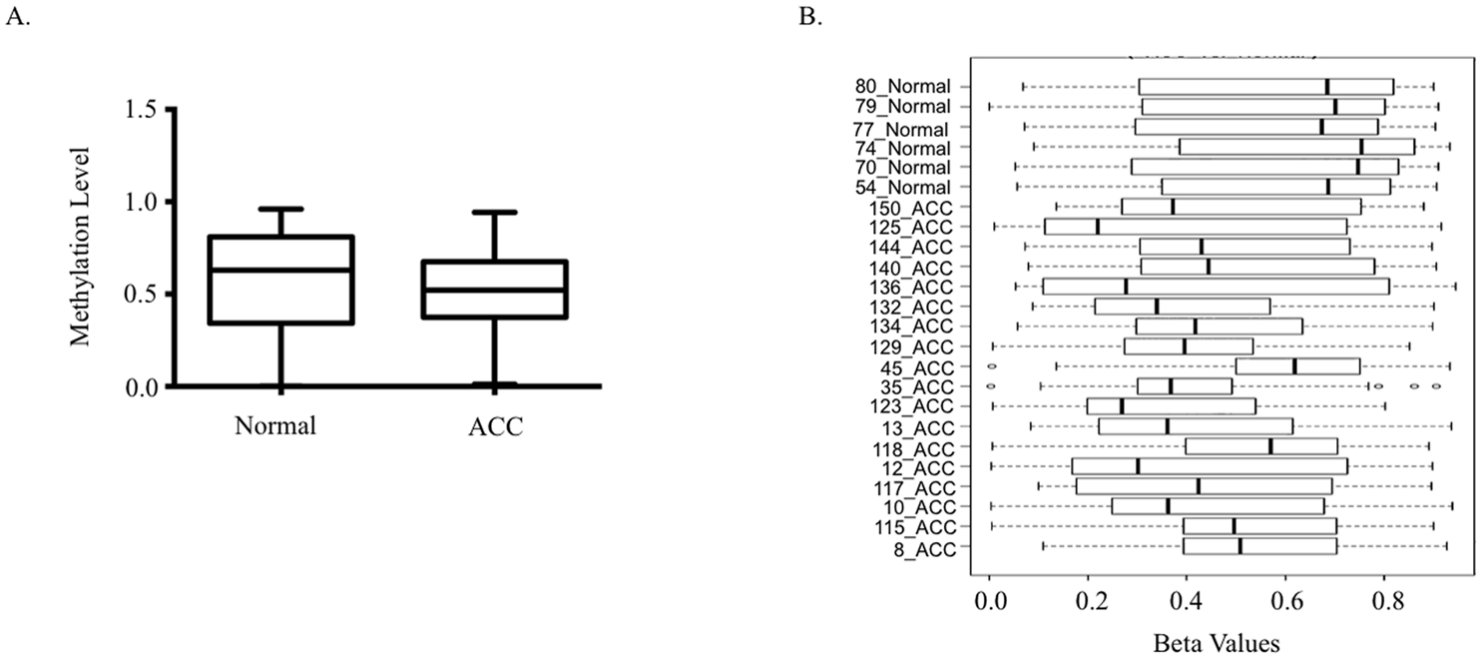
Differential Methylation Analysis comparing ACC to normal adrenal. **A**) Box plot demonstrating overall lower median β value methylation levels for 1291 differentially methylated probes. **B**) Box plots showing β values of 1291 differentially methylated loci for each individual sample (6 normal and 18 ACC samples).

To assess global DNA methylation patterns in ACC tumors, we used the 450K-methylation platform to compare 18 ACC tumors with 6 normal adrenal gland samples. Overall, the analysis revealed 1291 differentially methylated CpG loci (DML) encompassing 629 unique genes **(**S1 Table**)**. Beta values were used for generating box plots to represent overall methylation levels across DML for ACC and normal. Median overall methylation was slightly lower in ACC (β = 0.51) than in normal adrenal (β = 0.67) (Fig 1A), indicating hypomethylation in ACC (p-value < 0.0001). Individual sample box plots of DML demonstrated a more homogenous distribution of methylation values in normal adrenal samples compared to ACC which displayed varying degrees of methylation between samples (Fig 1B). Of all DML, 475 were hypermethylated and 816 were hypomethylated. Next we examined the distribution of DML across chromosomes, plotting the distribution of hypo- and hyper-DML after normalization to chromosome length (Table 1). According to the analysis chromosomes 5, 7, 12, and 19 had the most hypomethylated loci and chromosomes 11, 17, and 19 had the most hypermethylated loci, with chromosomes 7, 12, and 19 having the most overall DML.

**Table 1:** Frequency of DML by chromosome after normalization to chromosome length.

Chromosome	Frequency Hypo	Frequency Hyper	Total Frequency
1	694	520	1214
2	403	391	794
3	597	247	844
4	723	196	919
5	1354	653	2007
6	758	387	1145
7	1883	652	2535
8	965	335	1300
9	204	184	388
10	978	383	1361
11	811	854	1665
12	1937	581	2518
13	450	125	575
14	510	349	859
15	393	534	927
16	861	510	1371
17	568	1242	1810
18	332	184	516
19	1998	2193	4191
20	549	411	960
21	539	60	599
22	56	449	505

**Fig 2:**
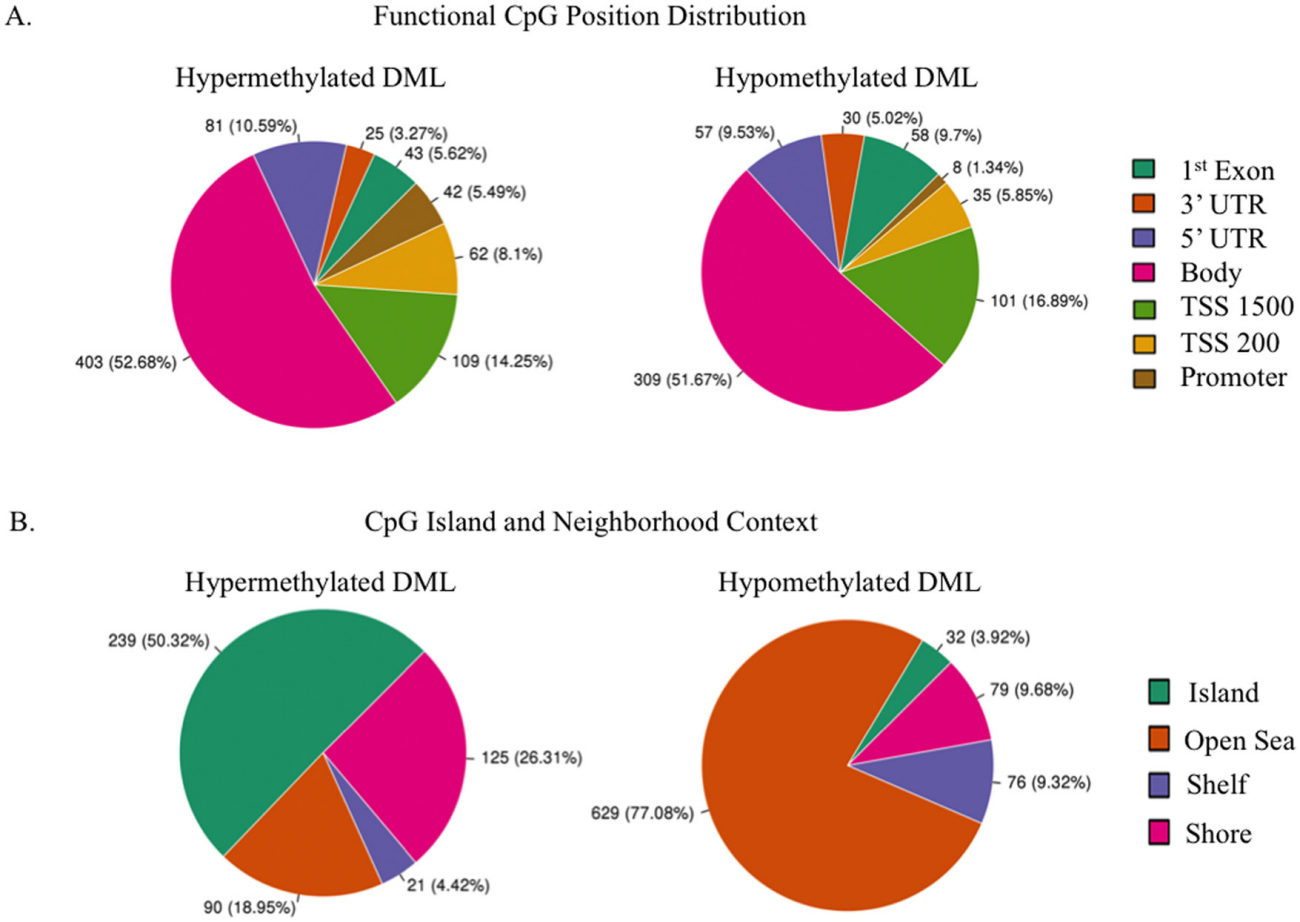
Frequency of differentially methylated regions according to functional and CpG Island contextual distribution. **A**) Pie charts demonstrate the frequency by which hyper or hypomethylated loci are distributed according to their functional position, including distance from transcriptional start site (TSS). **B**) Frequency of DML to be situated in a CpG islands, shores, shelves, or open sea. Neighborhood context is an indication of proximity to a CpG island.

Next we examined the regional and functional CpG distribution of DML in ACC. Functional distribution relates CpG position to: transcription start sites (TSS −200 to −1500 bp), 5′ untranslated region (UTR), exon 1 for coding genes, and gene bodies. Overall, the majority of probes (>50%) were situated in gene bodies, followed by ~15% of probes situated within 1500 bp upstream of TSS, with a very similar breakdown between hyper and hypo DML (Fig 2A).

Regional distribution of DML was assessed based on their proximity to the closest CpG island. In addition to islands, shores are 0–2 kb from CpG islands, shelves are 2–4 kb away, and open sea regions are isolated loci without a designation. When comparing the ACC samples to normal samples, we identified the majority of DML (55.7%) were in open sea, followed by islands (21%), shores (15.8%), and shelves (7.5%). In addition, we note that the majority of hypermethylated loci (50.3%) were located in CpG islands compared to the majority of hypomethylated loci being situated in open sea (77%) (Fig 2B). In contrast, of all hypomethylated loci, the smallest percentage (3.92%) was found in islands and the smallest percentage of hypermethylated probes (4.42%) were found in shelves.

**Fig 3:**
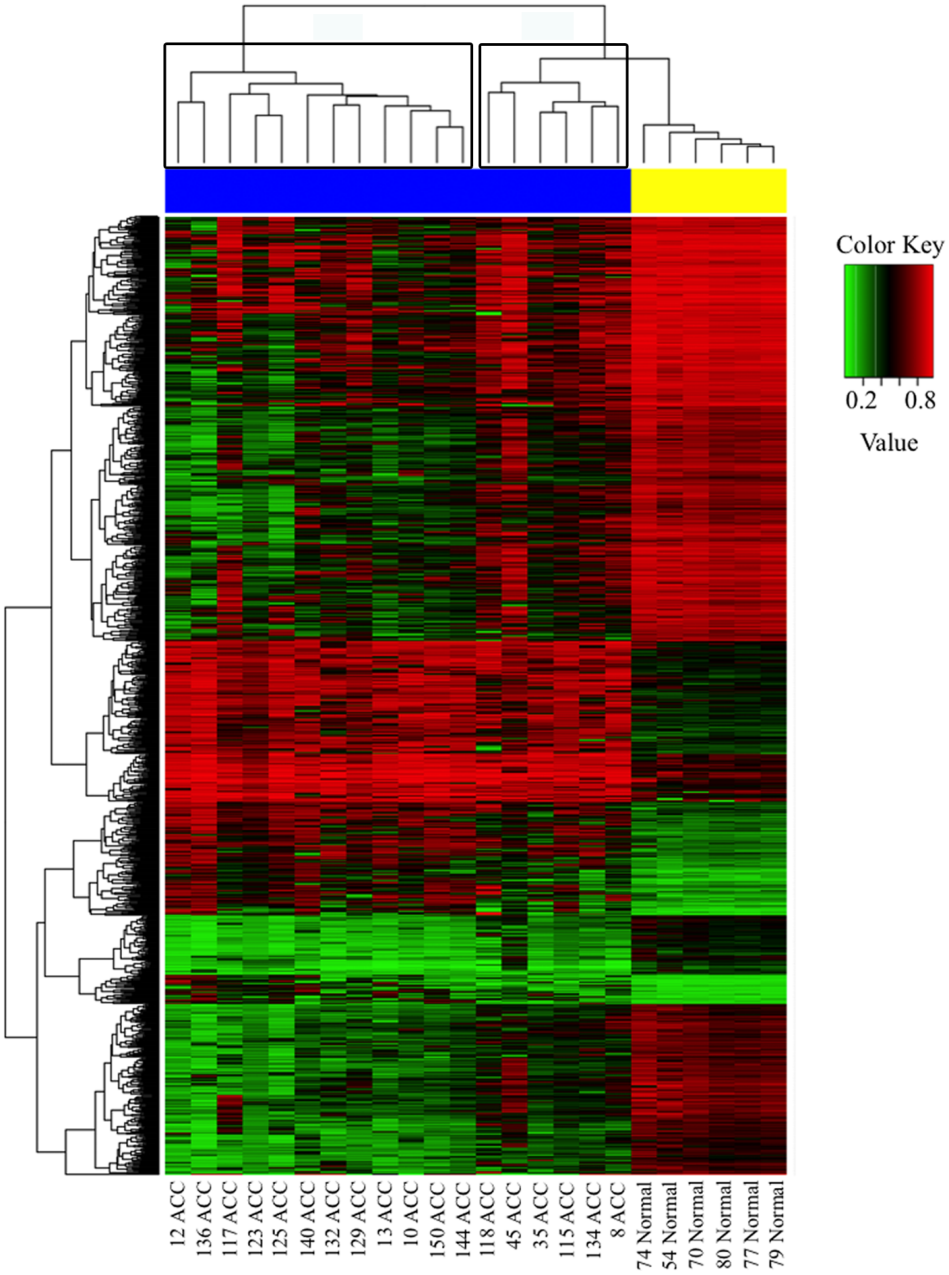
Unsupervised clustering analysis of normal and ACC samples using DML. Clustering analysis revealed separation of ACC and normal samples. Samples are on the horizontal axis: normal samples are shown with a yellow bar and ACC samples are shown with a blue bar.

**Fig 4:**
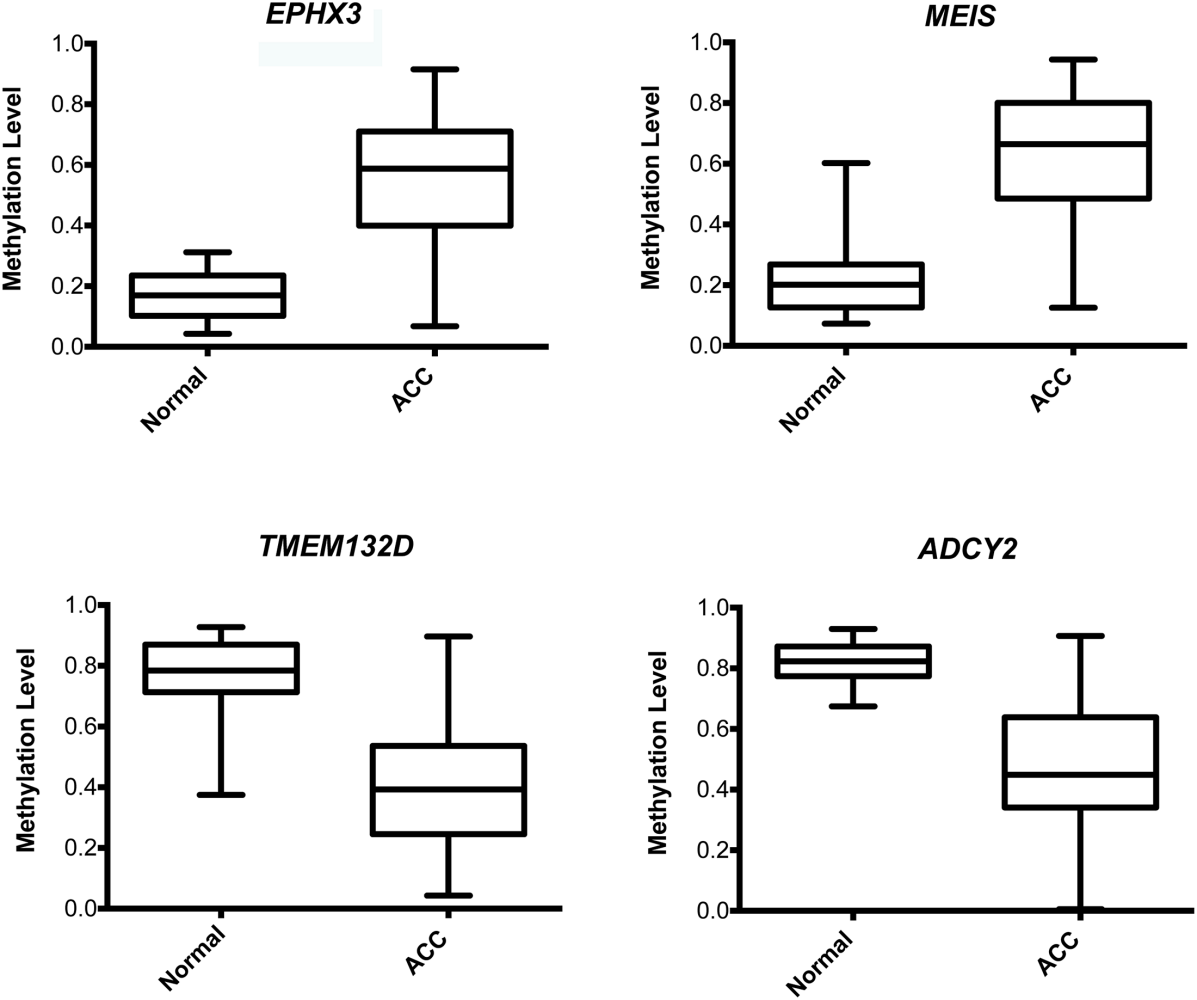
Box plots display examples of genes significantly hyper or hypomethylated in ACC. *EPHX3* and *MEIS* genes are significantly hypermethylated in ACC compared with *TMEM132D* and *ADCY2* which are significantly hypomethylated.

We next performed unsupervised clustering analysis (Euclidean distance, Complete hierarchical clustering methods) of DML and demonstrated a distinct separation of normal and ACC samples with evidence of gene clusters that are preferentially hyper or hypomethylated in ACC (Fig 3). Among the genes most hypermethylated in ACC were the *EPHX3* and *MEIS* genes, with 20% and 9.2% of possible gene probes affected (Fig 4). Representative genes in the hypomethylated clusters are the *ADCY2* and *TMEM132D* genes, which affected 16.2% and 18.5% of probes (Fig 4).

### Identification of Methylation-Expression Correlations Using the Discretization Method

In a separate analysis, we examined methylation-expression correlations using a non-linear approach: referred to as the *discretization method*. This method relies on the fact that cancer samples can be categorized into two groups according to their relative methylation levels for each probe on the methylation array, enabling a higher number of correlations to be detected than by comparisons using linear methods. Samples were either considered *hypermethylated* (M) or *hypomethylated* (U) compared to a reference (average methylation levels from normal samples in this study) and statistically significant gene expression differences between samples in the *M* and *U* groups would suggest a methylation-expression correlation at a given locus. By applying this binary discretization method, we identified 763 unique CpG loci (550 unique genes) with methylation-expression correlations. On average, there were 9 samples in the *M* group and 5 samples in the *U* group. There were 319 loci with positive correlations and 444 loci with negative correlations. All CpG methylation/expression correlations from this discovery cohort are shown in S2 Table.

In order to validate the findings of the current study in an independent cohort of samples, we downloaded RNA-seq expression data and matching DNA methylation data from TCGA portal for 78 ACC samples. This sample size not only allowed us to test correlations using our modified binary discretization method, but also provided the opportunity to utilize the ternary method as well. Due to absent/missing data in the TCGA set, 468 probes were available for binary testing of coordinated expression. Additionally, some probes lacked sufficient class size (M and U), thus leaving 433 probes for ternary grouping out of 763 unique probes reported in S2 Table. In binary grouping, 164/468 (35%) probes (S3 Table) showed significant correlation (p-value < 0.05) with corresponding gene expression values, and in ternary grouping 154/433 (35.6%) probes (S4 Table) showed significant correlation (p-value < 0.05).

### Alterations in TP53 and WNT Signaling Pathways

In order to identify biological concepts enriched in S2 Table, we both visually inspected the list to identify obvious patterns and we submitted the gene list to Core Analysis workflow in IPA (Ingenuity^®^ Systems). Significant findings were sorted based on p-values to identify the most distinguishing categories representing epigenetically regulated genes in ACC. In brief summary, the most significant Diseases and Disorders associated with the genes in S2 Table was Cancer with 471 genes represented from our list (S5 Table). The 5 top Molecular and Cellular Functions were Cellular Growth and Proliferation, Cell Morphology, Cellular Assembly and Organization, Cellular Function and Maintenance, and Cell Death and Survival (S6 Table).

**Fig 5:**
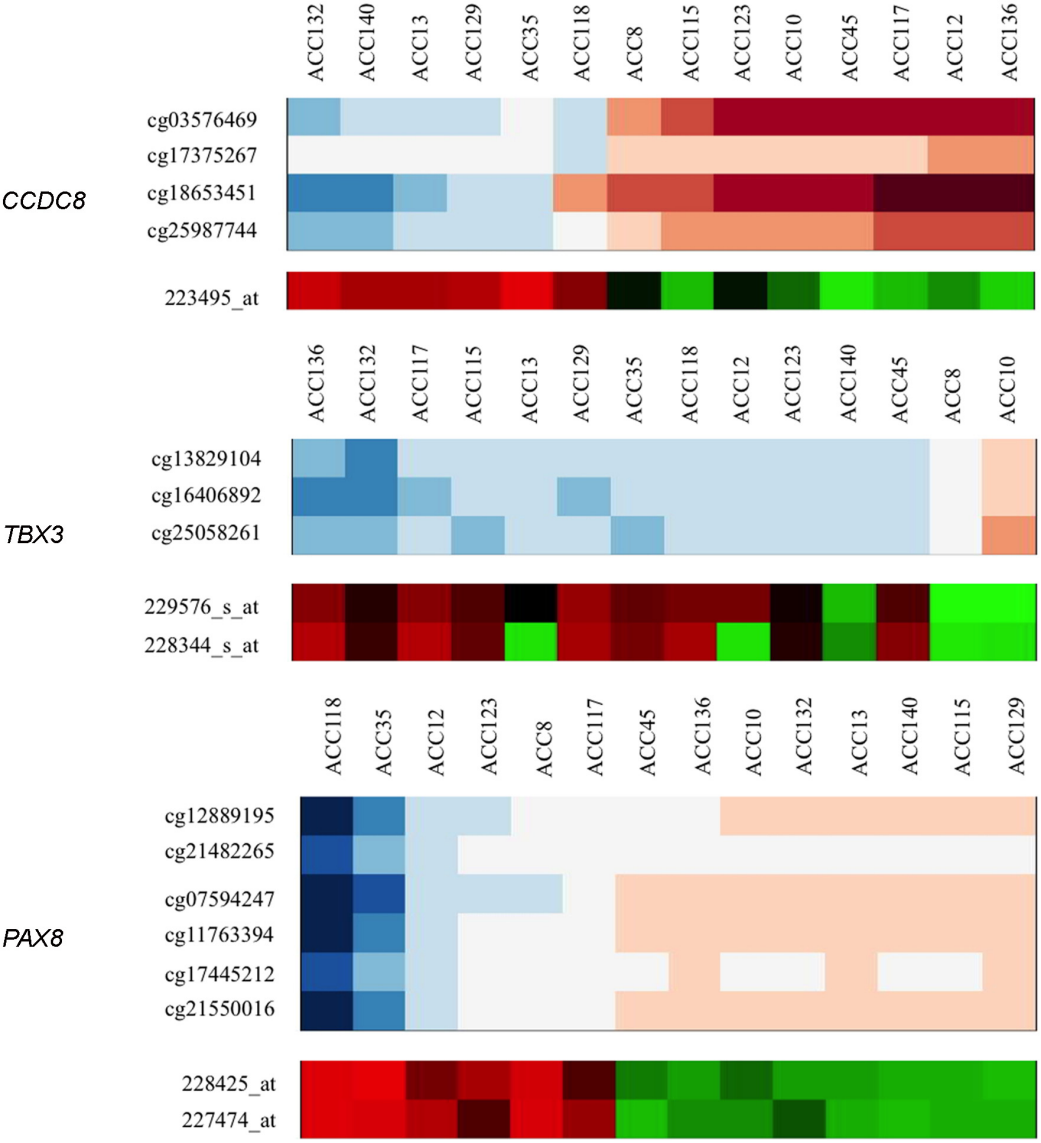
Methylation/expression correlations according to discretization method. For each gene, the upper heatmap represents the log2 methylation values for 14 ACC samples each normalized to the average of 6 normal adrenal samples. Log2 methylation ratios >0 represent hypermethylation and <0 represent hypomethylation. The lower heat map shows expression of z-transformed expression levels, where a value 0 indicates no expression change compared to average expression level of 14 ACC samples. Three genes with negative correlations were selected for visualization: *CCDC8* (TP53 pathway), *TBX3* (WNT pathway), and *PAX8* (other known cancer gene, involved in invasion and migration). Samples with higher methylation (peach-maroon) had lower expression (green). Samples with lower methylation (blue) had higher expression (red). Only the correlated methylation loci and expression probes are shown, and samples are organized by their discretization classification of M or U for each gene.

**Fig 6:**
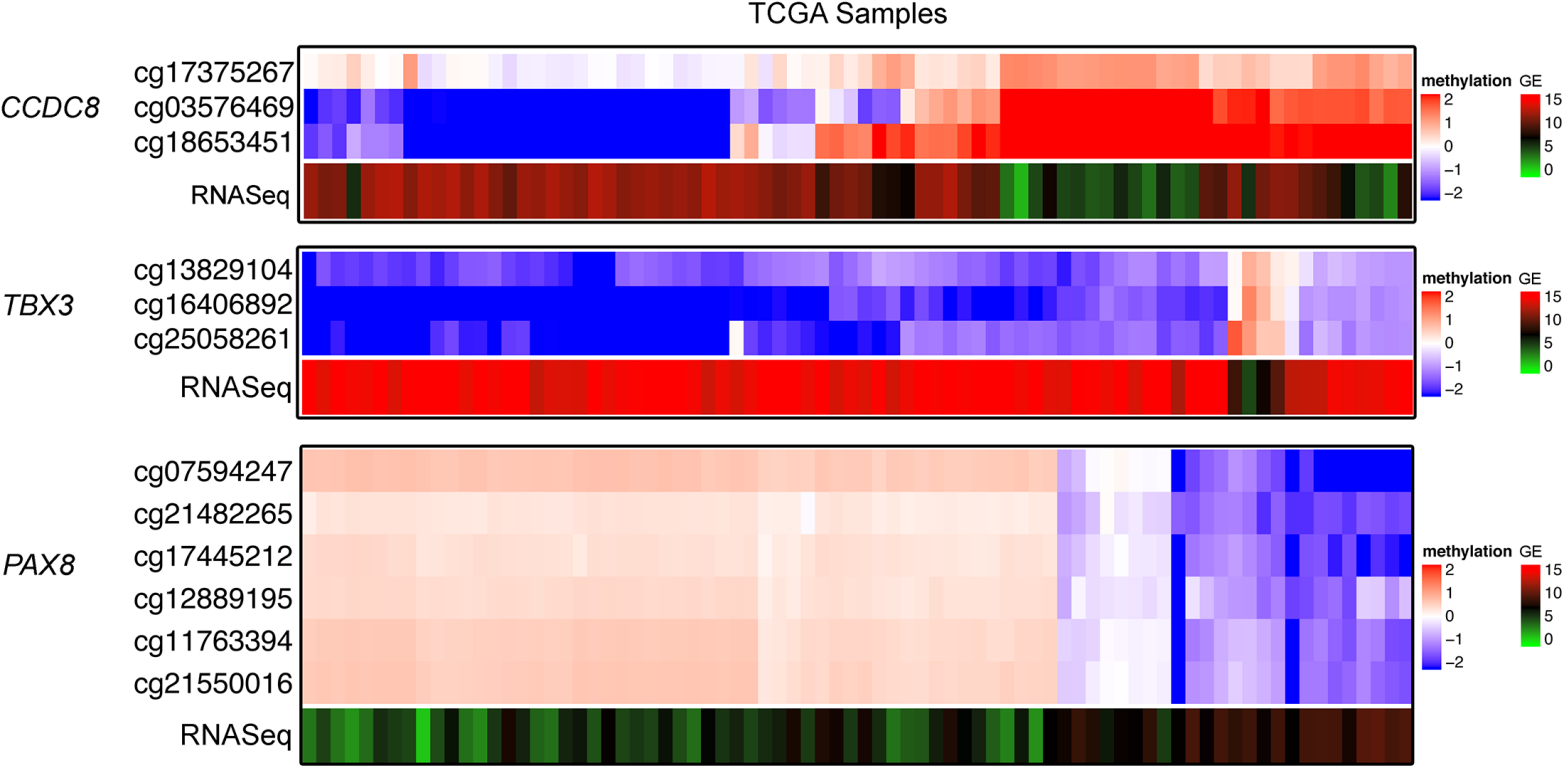
TCGA Validation: Methylation/expression correlations according to discretization method. For each gene, the upper heat map represents the log2 methylation values (beta) for 78 TCGA ACC samples each normalized to the average of 6 TGen normal adrenal samples. Log2 methylation ratios >0 (red) represent hypermethylation and <0 (blue) represent hypomethylation. The lower heatmap shows gene expression of corresponding TCGA ACC samples, log2 (TPM+1). The same three genes used for TGen ACC study (discovery) were used for visualization: *CCDC8* (TP53 pathway), *TBX3* (WNT pathway), and *PAX8* (other known cancer gene, involved in invasion and migration). Samples with higher methylation (red) had lower expression (green). Samples with lower methylation (blue) had higher expression (red). Only the correlated methylation loci and expression probes are shown, and samples are organized by similarity of methylation patterns between samples for each gene. As shown, all three genes show the same negative correlation between methylation status and gene expression as TGen ACC samples.

Using the discretization method, we identified a number of genes with methylation-expression correlations known to be involved in cancer, or specifically ACC pathogenesis. All of the genes we point out below were enriched in the Ingenuity Pathway Analysis under one of the categories mentioned above. Their pathway association and whether or not they were validated is noted on each of the tables below. Among this list of correlated genes are regulators of the TP53 pathway, such as: *SETD7*, *DYRK2*, *CCDC8*, *UBE2D1*, *RBM5*, *NDRG1* and *DUSP7* (Table 2, Figs 5 and 6, S2–S4 Tables). In each example, samples with low methylation had statistically significant higher levels of gene expression and samples with high methylation had lower levels of gene expression. *DUSP7*, *NDRG1*, *SETD7*, and *UBE2D1* were observed to be under-expressed in an independent data set comparing ACC mRNA expression to normal adrenal, with the under-expression agreeing with the observed hypermethylation/under-expression correlation [21].

**Table 2:** Significantly regulated genes in the TP53 pathway.

Gene	Methylation Discretization (high/low)	BH-corrected p-value	Direction of correlation	TCGA Validated (Binary/Ternary)	Alternate Validation Cohort (mRNA)	TP53 relationship
*CCDC8*	9/5	0.00003	-1	Yes/Yes	No	Tumor suppressor, co-factor in TP53-mediated apoptosis
*DUSP7*	12/2	0.0005	-1	NA	Yes (0.00001)	MAPK inactivator, TP53 target gene53 target gene
*DYRK2*	12/2	0.0004	-1	NA	No	Regulates TP53 stability
*RBM5*	12/2	0.0010	-1	NA	No	Regulates TP53 activity
*SETD7*	12/2	0.0005	-1	NA	Yes (0.00001)	Stabilizes TP53
*NDRG1*	11/3	0.0200	-1	NA	Yes (0.03)	Tumor suppressor, necessary for TP53-mediated apoptosis
*UBE2D1*	12/2	0.0300	-1	NA	Yes (0.000005)	TP53 ubiquitination and turnover

(NA = data not available for gene in TCGA dataset)

We also identified methylation/expression correlations in 4 WNT signaling-related genes; this is another pathway known to be associated with ACC tumorigenesis (Table 3, S2–S4 Tables). Samples displaying higher methylation of *PRDM5* and *DKK3*, which are both antagonists of WNT signaling, were accompanied by loss of mRNA expression and vice versa. Conversely, *TBX3* (a WNT target gene) was hypomethylated and overexpressed; these correlations were observed in both the original and TCGA datasets (Figs 5 and 6). *WNT3* (WNT ligand) was methylated and underexpressed in 11 samples and unmethylated and overexpressed in 3 samples. Loss of *DKK3* mRNA expression was validated in an independent, publicly available data set comparing ACC to normal adrenal tissue [21].

**Table 3:** Significantly regulated genes in the WNT pathway.

Gene	Methylation Discretization (high/low)	BH-corrected p-value	Direction of correlation	TCGA Validated (Binary/Ternary)	Independent mRNA validation (p-value)	WNT relationship
*TBX3*	2/12	0.0001	-1	Yes/Yes	No	WNT target gene
*PRDM5*	12/2	0.01	-1	Yes/Yes	No	Tumor suppressor, regulates WNT signaling
*DKK3*	11/3	0.04	-1	Yes/Yes	Yes (1.76E-10)	WNT antagonist
*WNT3*	11/3	0.02	-1	No/No	No	WNT ligand

### Other Cancer-Related Gene Alterations

The transcription factor *PAX8*, shown to be hypermethylated in other tumor types, largely demonstrated a negative correlation to gene expression, where hypermethylation correlated with reduced expression (Table 4, Figs 5 and 6, S2–S4 Tables). Negative methylation/expression correlations were also found for *HDAC4* (a chromatin modifier), *ERBB3* (a known oncogene), as well as *MARCKS* and *CXCR4*; genes known to induce tumor cell invasion and therapeutic resistance in other tumor types (Table 4, S2–S4 Tables). The role of IGF2 and IGF signaling as an oncogenic driver in ACC is also well described. Our data reveal a significant methylation/expression correlation for the *IGF2* gene. One probe (cg04057455) situated in the gene body correlated positively with two gene expression probes (S2 Table). The other probe (cg08986368) located in a CpG island correlated negatively with one gene expression probe. The end result was increased expression for samples with CpG island hypomethylation or gene body hypermethylation, which is consistent with the expected effects of methylation on gene expression.

**Table 4:** Significantly regulated genes associated with cancer.

Gene	Methylation Discretization (high/low)	BH-correctedp-value	Direction of correlation	TCGA Validated (Binary/Ternary)	IndependentmRNA validation (p-value)	Proposed Function
*PAX8*	9/5	0.05	-1	Yes/Yes	Yes (0.0002)	Transcription factor
*HDAC4*	12/2	0.009	-1	No	Yes (0.00001)	Chromatin modifier
*MARCKS*	2/12	0.03	-1	No	Yes (0.00002)	Cell invasion and therapy resistance
*CXCR4*	3/11	0.03	-1	Yes/Yes	No	Cell invasion and metastasis
*ERBB3*	12/2	0.0002	-1	Yes/Yes	Yes (0.006)	Oncogene

## Discussion

The purpose of the current study was to analyze global DNA methylation patterns in ACC tumors compared to normal adrenal tissue and to correlate DNA methylation changes with mRNA expression. Our study demonstrated global hypomethylation in ACC compared to normal adrenal tissue. Global genomic hypomethylation has been observed across numerous cancer cell types [22, 23], including malignant adrenal tumors when compared to benign or normal adrenal tissue [14]. Consistent with previous global methylation analyses, including ACC, hypomethylation was most frequent in ‘open seas’, away from the CpG islands, and hypermethylation events occurred most commonly in CpG islands [15].

To identify significant correlations between methylation and gene expression in ACC, a discretization method was employed. This non-linear method for identifying correlations is more sensitive than linear methods because it relies on subsets of samples having opposite methylation trends [16]. With this method we identified 550 genes with significant negative or positive correlation of methylation and gene expression. The genes identified using this method implicate pathways known to be perturbed in ACC such as TP53, WNT, and IGF2 signaling. In addition, classical tumor suppressor, other cancer related, and novel genes not previously implicated in ACC were also identified. The *TP53* tumor suppressor is mutated in more than 50% of all tumors. Genetic alterations in *TP53*, including mutations (16%) [7] and epigenetic silencing (rarely seen) [24], occur in a lower percentage of ACC tumors. Our recent work in ACC demonstrated significant differential expression of genes involved in TP53 canonical signaling [8]. Gene relationships to *TP53* were determined by their inclusion in the Gene Set Enrichment Analysis (GSEA) gene sets PID_P53DOWNSTREAMPATHWAY, PID_P53REGULATIONPATHWAY, and/or other reported evidence of TP53 binding or modulation of activity. In the current study, we observed several genes including *SETD7* [25], *DYRK2* [26], *CCDC8* [27], and *UBE2D1* [28] which are known to control TP53 protein stability and turnover through post-translational modifications and were epigenetically regulated in ACC tumors. Thus, while *TP53* is not differentially methylated or frequently highly mutated in ACC tumors, the methylation status of genes known to modulate TP53 stability and activity may provide an explanation for the observed TP53 deregulation.

Another pathway associated with ACC tumorigenesis is the WNT signaling pathway. WNT signaling has been aberrantly detected in ACC tumors evidenced in part by frequent mutations in the *β-catenin* gene [7, 29]. Discretization analysis also revealed epigenetic regulation of WNT signaling-related genes in ACC. PRDM5 is a putative tumor suppressor through repression of WNT signaling, and known to be frequently silenced due to methylation across a number of other tumor types [30, 31]. We observed both promoter and gene body methylation, which correlated with altered mRNA expression. The known WNT antagonist DKK3, normally expressed in the human adrenal cortex [32], is under-expressed in childhood adrenocortical tumors [33]. DKK3 affects apoptosis and cell proliferation [34], and our study now points to CpG methylation of *DKK3* as a possible mechanism for gene deregulation in ACC tumors [21].

Using the discretization method, we also identified a number of putative tumor suppressor genes whose methylation correlated with decreased gene expression. MARCKS, a substrate for protein kinase C phosphorylation, displays tumor suppressive roles in multiple cancer types[35]; our study is the first report of *MARCKS* epigenetic silencing in ACC. Similarly, hypermethylation of *PAX8* correlated with downregulation and low methylation correlated with increased expression. Elevated levels of *PAX8* have been seen in several tumor types and epigenetic silencing has been observed in squamous cell lung cancer [36]. The prognostic and biological implication of PAX8 expression is likely tissue and organ specific.

In conclusion, we have demonstrated that differential DNA methylation and expression correlation of ACC tumors and normal adrenal tissue identified potential genes and pathways with relevance to underlying biology and potentially patient outcome. Although this study had a limitation in small sample size that precluded a statistical evaluation of patient outcome data, the results were validated by analyzing the TCGA ACC dataset. Future work will focus on molecular and biological subclassification of ACC tumors with DNA methylation and making correlations to patient outcomes. Lastly, correlations of methylation and gene expression by the discretization method identified, for the first time, epigenetic modulation of genes involved in TP53 stability and function, WNT signaling, and tumor suppressor genes not previously associated with ACC.

## Supporting Information

S1 Table List of Differentially Methylated Probes. (XLSX)

S2 Table List of Methylation-Expression correlations according to discretization method. (XLSX)

S3 Table List of TCGA Methylation-Expression correlations according to published Binary method. (XLSX)

S4 Table List of TCGA Methylation-Expression correlations according to published Ternary method. (XLSX)

S5 Table Ingenuity Pathway Analysis (IPA) results for binary discretization correlated gene list. (PPTX)

S6 Table Top 5 molecular and cellular functions for associated gene list as determined by IPA analysis. (XLSX)
