# Supplementary material for: Pathway Implications of Aberrant Global Methylation in Adrenocortical Cancer
Source: PLoS One. 2016 Mar 10;11(3):e0150629. doi: 10.1371/journal.pone.0150629 (PMC4786116; doi:10.1371/journal.pone.0150629)
Supplement: S5 Table — (PPTX) [file pone.0150629.s005.pptx]

## Slide 1
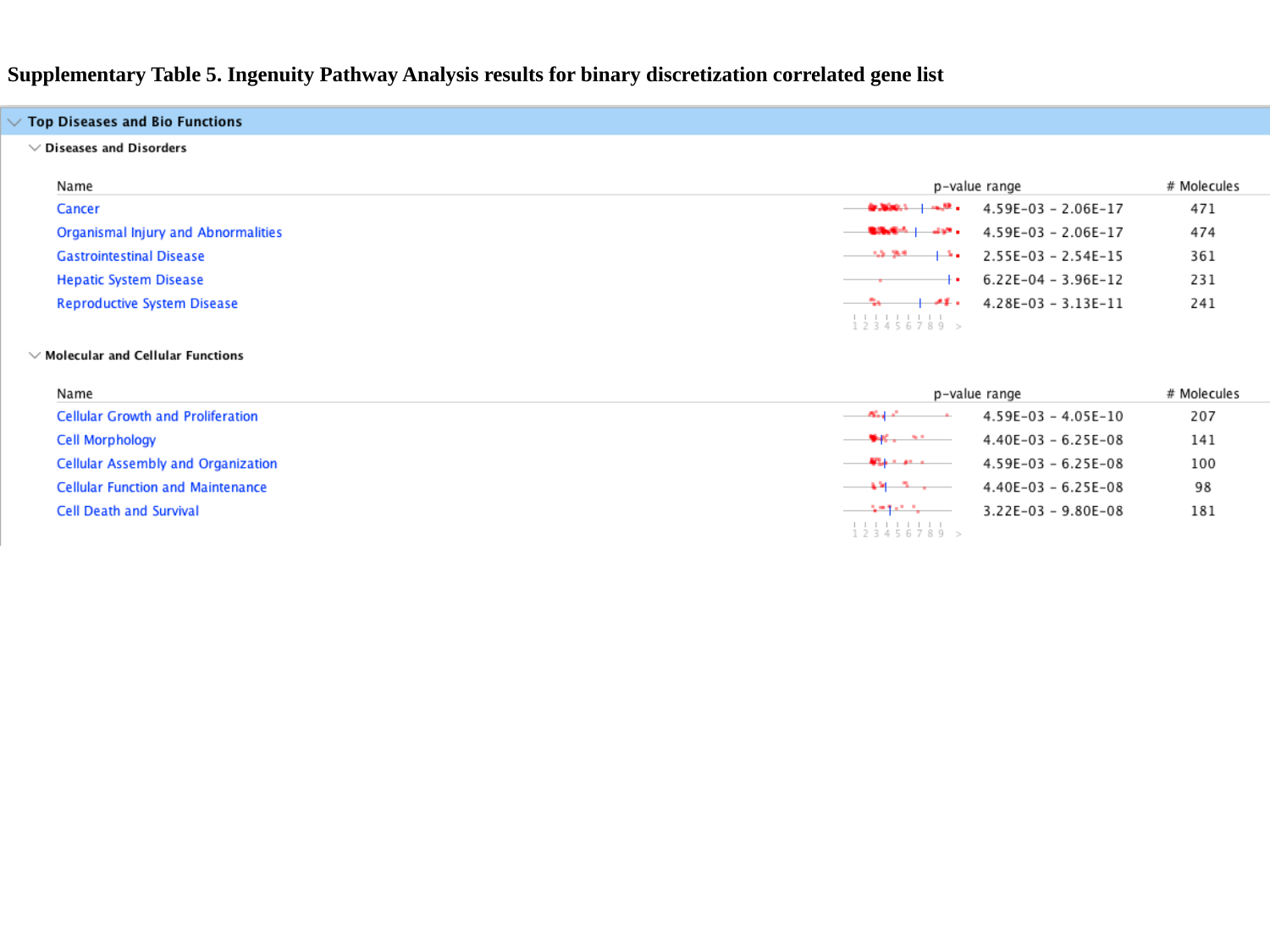

Supplementary Table 5. Ingenuity Pathway Analysis results for binary discretization correlated gene list
